# On the Causes and Treatment of Goitre

**Published:** 1843-06

**Authors:** 


*On the Causes and Treatment of Goitre.*—A communication was read to the French Academy of Sciences, August 1, from Pascal, on the local influences which assist in the development of goitre, and on the use of ferruginous mineral waters in preventing and curing this affection. From the observations of Bénédict, Fodéré, and Roulin, it appears, 1st, That the stagnation of humid air, whether cold or warm, eminently predisposes to this disease, and that all the moist gorges of the mountains towards the north are more particularly the localities in which bronchocele exists to the greatest extent. 2d. That the use of impure cold water, as the habitual beverage, is, if we may say so, the exciting cause of the affection in those places already predisposed. The water from wells, cisterns, and pumps, is said to be the most injurious.

Pascal then directed attention to the following fact:—At about three leagues from Metz are three villages, named Pierre Villers, Rombas, and Villers les Rombas, all of which are similarly situated as regards hygiène, with the exception that Villers had a chalybeate spring, which was generally used by the inhabitants for cooking and drinking. Now fifty years ago, while goitre attained a large size, and was very common in the two other villages, it was unknown in Villers; and it was observed, that those who came to reside in it from Pierre Villers and Rombas, got cured if they were already affected with the disease, or if they had it not at the commencement of their residence, continued free from it. From this circumstance we cannot but admit that the chalybeate was the cause of the inhabitants of Villers not being affected with bronchocele. Since the above period goitre has almost entirely disappeared from the other two localities, owing to the improvements that have taken place in the condition of the atmosphere, in the houses of the villages, and in the food of the inhabitants.

From these facts we may conclude, 1st, That notwithstanding the humidity and stagnation of the atmosphere, the use of a chalybeate water prevents the development of goitre, and cures it when it is formed; and, 2d, That even when no ferruginous spring exists, amelioration in hygiène greatly counteracts the predisposition of the inhabitants to be affected with bronchocele.—*Amer. Journ. Med. Scien.*, from *Lond. and Edin. Month. Journ. of Med. Sci.*, Dec. 1842.

